# On the Use of Sensor Fusion to Reduce the Impact of Rotational and Additive Noise in Human Activity Recognition

**DOI:** 10.3390/s120608039

**Published:** 2012-06-11

**Authors:** Oresti Banos, Miguel Damas, Hector Pomares, Ignacio Rojas

**Affiliations:** Department of Computer Architecture and Computer Technology, University of Granada, C/Periodista Daniel Saucedo Aranda s/n, Granada E18071, Spain

**Keywords:** activity recognition, rotational noise, additive noise, metaclassifier, accelerometer, sensor fusion

## Abstract

The main objective of fusion mechanisms is to increase the individual reliability of the systems through the use of the collectivity knowledge. Moreover, fusion models are also intended to guarantee a certain level of robustness. This is particularly required for problems such as human activity recognition where runtime changes in the sensor setup seriously disturb the reliability of the initial deployed systems. For commonly used recognition systems based on inertial sensors, these changes are primarily characterized as sensor rotations, displacements or faults related to the batteries or calibration. In this work we show the robustness capabilities of a sensor-weighted fusion model when dealing with such disturbances under different circumstances. Using the proposed method, up to 60% outperformance is obtained when a minority of the sensors are artificially rotated or degraded, independent of the level of disturbance (noise) imposed. These robustness capabilities also apply for any number of sensors affected by a low to moderate noise level. The presented fusion mechanism compensates the poor performance that otherwise would be obtained when just a single sensor is considered.

## 1. Introduction

The recognition of the human daily tasks has attracted much attention in the recent years. In particular the analysis of the human physical behavior, mainly described through the kinematics of the body, has been shown very interesting application areas. Sport assistance [1], manufacturer industry [2] or user-computer interaction [3] are examples of potential fields where the usefulness of such recognition systems is clearly identified. Besides, some of these devices are being currently materialized in end-user products such as Microsoft Kinect or Nintendo Wiimote, which provide impressive revenues (The Kinect sold 8 millions units in the first 60 days since its release on 4th November 2010, earning it the Guinness World Record of the “Fastest selling consumer electronic device”, and 18 million units of the Kinect sensor had been shipped as of January 2012) in the video games industry. However, the potential of the daily living activity recognition does not reduce its application scope to industrial or entertainment scenarios. Health care and assisted living are among the most promising and challenging application fields of activity recognition systems.

In the last decades representative global institutions have emphasized on the need of a change into the traditional health care models in order to avoid a foreseeable future collapse [4]. Primal factors such as a better nutrition, greater hygiene or more efficient clinical treatments determine a tendency on an aging population in developed countries. This translates into an eventual higher number of potential patients. The consequence is a critical rise on the demand of health care specialists and caregivers, infrastructures and equipment, which is hardly affordable by administrations and governments. Important changes should be accomplished in order to guarantee a sustainable health care model as well as to ensure the stability of the welfare state. The solution is not trivial, requiring the implication of different social, economic and political classes. Nevertheless the change is on as demonstrates the huge forth effort the countries and institutions are putting at present [5]. At any rate, what is clearer over time is that new methodologies should be considered in order to share the burden of the high dimensional problem addressed.

Two of the most interesting concepts in the envisioned health care model are subject specialization and proactivity. Specialization refers to a continuous personalized understanding of the individuals which may result in a more adequate treatment. The aim of proactivity is avoiding, whenever possible, the eventual treatment through the prevention of risk conducts that lead to most of the prevalent diseases. As an example, it is well known that sedentary conducts potentially increase the probability of suffering a chronic non-communicable disease (e.g., cancer, heart failure and diabetes).

The continuous monitoring of the subject's daily physical activity may be valuable to assess the individual's status in the short and long-term. Traditional approaches are sufficient neither in terms of continuity nor reliability. For example, sporadic doctor's visits limit the monitoring continuity and subject self-annotations constraint the reports reliability [6]. The ideal case would be having an expert who continuously monitors the subject without requiring direct human intervention. Activity recognition enables the collection of user information via strategically-deployed autonomous systems. Moreover, collected data support decision making. Thus, activity recognition is a potential solution for patient continuous monitoring. In addition, retrieved information might also allow physicians and researchers to gain insight about the diseases' origin and the effect of the provided treatments.

Activity recognition is a highly complex problem due to the several different factors that must be considered. Some of them are directly related to the subjects such as the age, gender, weight or height, introducing substantial differences among the individuals' execution styles. Moreover these conducts may be potentially disturbed when the subject suffers from physical or cognitive disabilities and/or diseases. Ambient and context-related factors also complicate the recognition task (e.g., we expect different motion styles when a barefoot subject walks on the grasp than when walking with ski boots). The system designer has to deal with the major part of these issues, however there exist other kind of problems particularly related to the sensor modalities used.

The use of cameras has been comprehensively studied in activity recognition (an extensive review is presented in [7]). However, there exist some drawbacks which may determine them as an inconvenient solution. Beyond important issues directly related to the end-user such as privacy, or particular technological challenges as occlusions, light absence or noise, one of the main problems for the use of cameras is the availability of such systems in real scenarios. Daily living activities do not solely circumscribe to households or buildings but also to outdoor environments where it might be complicated to have such sensor modalities. For example, activities such as hiking, cycling or skiing might be difficult to monitor with cameras in a realistic context. Since the aim is to perform a continuous monitoring of the individual's behavior, the system should be always available to monitor the subject under any circumstances.

The problems with the use of cameras are solved through the use of sensors which are permanently on the subject (on-body or wearable sensors). Inertial sensors are the most used modality to support the measurement of the body kinematics. One of the main problems on their use is about the obtrusiveness and discomfort experienced by the subject when wearing them. Nevertheless the latest advances in sensor miniaturization and integration permit us to envision a new generation of wearable devices which may be potentially integrated into the textiles (smart clothes) [8]. This constitutes a break-through in the race of developing realistic systems for the daily living monitoring.

Due to the aforementioned “wearability” issues, several activity recognition studies have focused on the reduction of the number of required sensors. Their aim is relying on one sole unobtrusive sensor [9]. Even assuming that a high level of performance is achieved, which is questionable when a relevant set of activities are targeted, these approaches present a lack of robustness against sensor failures or setup changes. Indeed, a generalized idea in activity recognition is considering that the sensor environment remains identical during runtime to the one foreseen at design-time. However, the user's daily living experience normally contradicts this assumption. Sensors may fail, run out of battery or their deployment may significantly change due to an unintentional incorrect sensor placement as a consequence of everyday use.

Recent research studied the issues related to prime inertial sensors anomalies as displacements, rotations and degradations. Kunze *et al.* described a first attempt to self-characterize sensors' on-body placement [10] and orientation [11] from the acceleration analysis during walking. They also demonstrated the effect of rotations and displacement in accelerometers, and proposed a way to partially deal with them through the use of additional sensor modalities [12]. These heuristic methods are coupled to the assumption that the user performs the specific activities required at some point, which nevertheless might not always be guaranteed. Foerster *et al.* [13] studied the possibility of system self-calibration through the adjustment of the classifier decision boundaries. This supports tracking the changes experimented in the feature space due to the sensor displacement. Similarly in [14] the authors proposed a method to compensate the data distribution shift caused by sensor displacements through the use of an expectation-maximization algorithm and covariance shift analysis.

The use of sensor fusion appears as an interesting approach to deal with sensor anomalies. A key advantage is that identifying the failure sources is not required to compensate the associated errors in the activity recognition process. Just a few previous works addressed this problem in this regard. Zappi *et al.* [15] show a significant tolerance increase by using a large set of sensors in combination with majority voting or naive Bayes decision fusion models. A more sophisticated model is presented in [16] attempting to detect anomalies and potential affected sensors in order to remove them from the sensor ecology.

In this work we further analyze which body sensor locations are more sensitive to sensor rotations, displacements or possible degradations. These disturbances are modeled through rotational and additive synthetic noise. In order to cope with the critical recognition performance drop that single sensor-based approaches may suffer when disturbed, we propose the use of a ranking fusion method which significantly enhances the robustness properties of the activity recognition systems.

The rest of the paper is structured as follows. Section 2 briefly describes the classical activity recognition methodology and explains the suggested fusion model. Section 3 introduces the sensor disturbance models as well as the results obtained for the individual sensor and fusion approaches. The results are subsequently discussed in Section 4 and our final conclusions are summarized in Section 5.

## 2. Activity Recognition Methods

In order to deal with most of the activity recognition issues a general methodology is normally proposed. A set of nodes (sensors) usually delivers raw unprocessed signals which represent the magnitude measured (e.g., acceleration). The registered information may be disturbed by electronic noise or other kind of artifacts. Depending on whether a certain information loss is tolerated, sometimes the signals are pre-processed through a filtering process [17,18]. In order to capture the dynamics of the signals these are partitioned in segments of a fixed or variable size. Different techniques are depicted for that purpose, mainly based on windowing [19] or event-activity based segmentation [20]. Subsequently a feature extraction process is carried out to provide a handler representation of the signals for the pattern recognition stage. A wide range of heuristics [21], time/frequency domain [22,23] and other sophisticated mathematical and statistic functions [24] are commonly used. The feature vector is provided as input of the classifier, ultimately yielding the recognized activity or class to one of the considered for the particular problem. An extensive topical review of the activity recognition classical methodology can be seen in [25].

In order to increment the reliability of the recognition system, a fusion model that combines the decisions of each individual sensor is proposed. The model takes in the main advantages of the hierarchical decision and majority voting models. The idea is giving all the entities the opportunity to collaborate on the decision making, but ranking the relative importance of each decision through the use of weights based on the classification entities performance.

**Figure 1.**
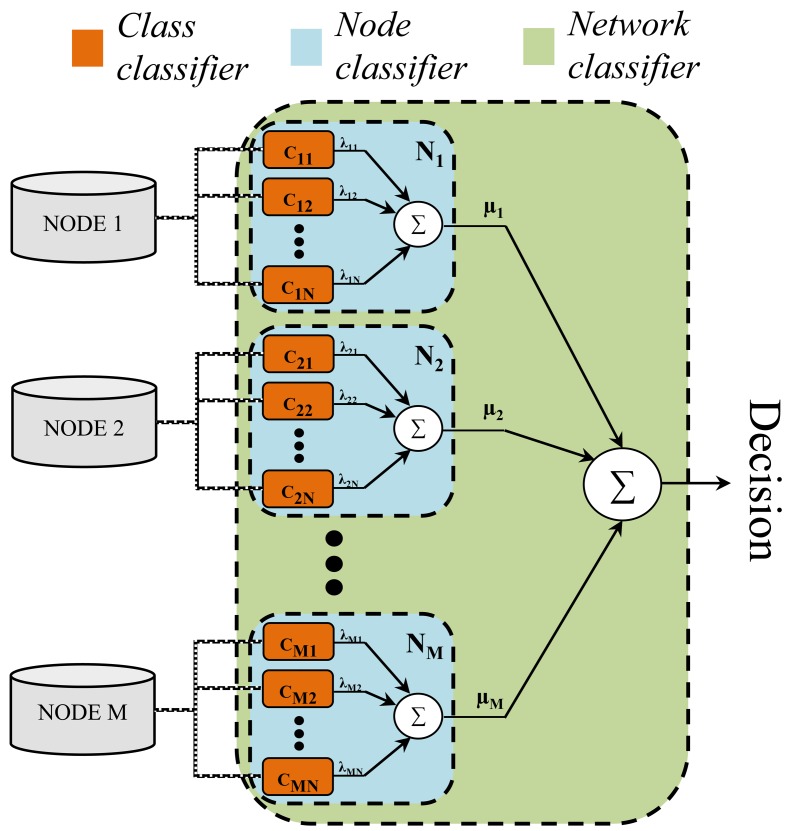
SWNC general structure for a problem with N classes and M nodes.

The proposed model, in advanced called Sensor Weighted Network Classifier (SWNC), is composed by three classification levels or stages (see Figure 1). Let us consider an scenario with *M* nodes of information (sensors) and *N* classes (activities). The first layer of the hierarchy is composed by a set of *M* by *N* base or “class classifiers” (*c_mn_*). They are binary classifiers specialized in the insertion/rejection of the class *n* by using the data acquired from the *m-th* node. Each one applies an one-*versus*-rest strategy (other approaches such as the one-*vs.*-one may be similarly applied but here the one-*vs.*-rest is particularly recommended in order to reduce the number of classification entities), allowing for the use of any kind of classification paradigm. The second level is defined by *M* sensors or “node classifiers” (*N_m_*). Node classifiers are not machine learning-type entities, but decision making models. Their structure is defined through several base classifiers as it is shown in Figure 1, which decisions are combined through a class-dependent weighting fusion scheme. This is replicated for the next level, leading to a complete model constituted by the fusion of the weighted decisions yielded by each node classifier.

A process consisting of a few main steps is carried out to infer the complete SWNC. The process starts by evaluating the individual accuracy of each class classifier. A *p*-fold cross validation is suggested for accomplish this task. The whole process is then repeated for each node. Considering the average accuracy rates ( $\bar{R_{mn}}$ for the node *m* and the class classifier *n*) as a reliable measure of the performance capabilities of each classifier, the weight for each base classifier is obtained as follows:

(1) $$\lambda_{mn}=\frac{\bar{R_{mn}}}{\sum_{k=1}^{N}\bar{R_{mk}}}$$

These weights represent the importance that each base classifier has on the node classifier decision scheme. At this point a specific voting algorithm is considered to combine the decisions yielded by each class classifier on a single node level decision. For a node *m*, given a sample *x_mk_* (described through the corresponding feature vector) to be classified and being *q* the class predicted by the classifier *c_mn_*, if such class belongs to the class of specialization (*q* = *n*), the classifier sets its decision to ‘1’for the class *n* and ‘0’ for the rest of classes. The opposite is made for (*q* ≠ *n*). The decision of the classifier *n* for the class *q* is given by (∀ {*q, n*} = 1,…, *N*):

(2) $$y_{nq}\;(x_{mk}\;)=\{\begin{array}{cc} \begin{array}{l} 1,x_{mk}\;\text{classified as}\;q\\ 0,x_{mk}\;\text{not classified as}\;q \end{array} & \left(\forall q=n\right)\\ \begin{array}{l} 1,x_{mk}\;\text{not classified as}\;q\\ 0,x_{mk}\;\text{classified as}\;q \end{array} & \left(\forall q\neq n\right) \end{array}$$

Now the output of the *m-th* node classifier can be computed as follows:

(3) $$O_{mq}\;(x_{mk})=\psi (\lambda_{mn}y_{nq}\;(x_{mk}))=\sum_{n=1}^{N}\lambda_{mn}y_{nq}\;(x_{mk})$$

The class predicted for the *m-th* node classifier (*q_m_*) is the class *q* for which the node classifier output is maximized:

(4) $$q_{m}=\underset{q}{\arg\;\max}\;(O_{mq}(x_{mk}))$$

At this stage the node level is completely defined. Every node classifier can be independently used but fusion or combination of nodes has been devised to be a more robust and efficient solution. Consequently, the complete process described before is extended to a new hierarchy level, the network level classifier. A weight is calculated for each node by assessing first the average accuracy rates for each node classifier ( $\bar{R_{mn}}$). This is performed through a new cross-validation process for the already trained node classifiers. The weight for the node *m* is:

(5) $$\mu_{m}=\frac{\bar{R_{m}}}{\sum_{k=1}^{M}\bar{R_{k}}}$$

The output is calculated taking into account the individual outputs obtained for each node classifier. For a sample *x_k_* defined through the corresponding information obtained from each node (*x*_1_*_k_*,…, *x_Mk_*), the output is:

(6) $$O_{q}\;(x_{k})=O_{q}\;(\{x_{1k},\ldots ,x_{Mk}\})=\psi (\mu_{p}{Ο}_{pq}(x_{pk}))=\sum_{p=1}^{M}\mu_{p}{Ο}_{pq}(x_{pk})$$

Similar to Equation (4) the final class predicted *q* is obtained as:

(7) $$q=\underset{q}{\text{argmax}}\;(O_{q}(x_{k}))$$

At the end the SWNC is fully characterized through the trained base classifiers (*c_mn_*), the class level weights (λ*_mn_*) and the node level weights (*μ_m_*).

## 3. Results

### 3.1. Experimental Setup

Considering the objectives of this study an activity recognition dataset rich on classes, sensors and data is particularly required. One of the most used and cited is the presented in [26]. It comprises the acceleration data registered for twenty subjects aged 17 to 48 while performing a set of daily living activities. From the whole set the most representative nine are selected: walking, running, cycling, sitting, standing still, lying down, stretching, strength-training and climbing stairs. The movements were recorded through five biaxial accelerometers attached to the subject's right hip, dominant wrist, non-dominant arm, dominant ankle and non-dominant thigh respectively.

In order to preserve the static components of the measured acceleration the signals are not filtered out as sometimes a normal practice does. This information may be particularly interesting to analyze how the possible disturbances affect the recognition process specially for sedentary or quasi-static activities. The raw signals are subsequently partitioned in data windows of approximately 6 seconds as suggested in [26]. Indeed some authors argue the sliding window approach does not require pre-processing of the sensor signal [25], which also supports the procedure here described. Moreover, it should be highlighted the windowing mechanisms are ideally suited to real-time applications, normally the ultimate goal of the activity recognition system. The problem of the null class or unidentified activities is not addressed here. It is assumed an independent system works behind providing the start and end point of the considered actions. The segmentation is then accordingly applied to the data delivered by such system.

Taking into account the fusion structure presented in Section 2, different feature vectors may be provided for each binary classifier. Indeed, in order to increase the potential of the base level entities, the 10 best features (L = 10, see Figure 1) ranked from an original set of up to 861 features [24] are considered. For that search a feature selector based on the receiver operating characteristic is used [27].

We use a k-nearest neighbor algorithm (kNN) as classification paradigm which has been demonstrated to accurately perform in previous activity recognition problems [25,28]. The coefficient k is empirically obtained for each base classifier.

For the system evaluation, 70% of the whole dataset is reserved to train the model and the remaining 30% for testing (all the subjects are considered). The training partition is likewise split in three equally-sampled subsets respectively dedicated for the class classifiers training, *λ_mn_* assessment and *μ_m_* assessment. The initial distribution into both sets (train and test) is randomly defined for each of the 100 times the process is repeated, thereby ensuring the statistical robustness.

### 3.2. Rotational and Additive Noise Models

As was presented in Section 1, the main modifications with respect to the initial setup may be due to an incorrect sensor attachment normally translating in different impact rotations and displacements. Besides, there are other relevant anomalies as sensors uncalibration, battery failures or problems during the data delivery, which may affect the normal functioning of the recognition system. Here we model these issues through the so-called rotational (RN) and additive (AN) noise.

According to the physics of the rigid body, the sensors may experience a rotation in the 3-dimensional Cartesian space. The rotation implies a change on the sensor local frame of reference with respect to its original spatial distribution. The acceleration readings in this new coordinates system likely differ with respect to those expectable from a correct sensor placement. This normally leads to a different signal space. In order to obtain the corresponding signals an Euclidean transformation of the raw data is considered. The transformation is here modeled through a matrix (*c*() and *s*() represent the cosine and sine functions respectively) defined by the Euler angles (*φ_rn_ θ_rn_, ψ_rn_*) respectively representing the rotation along the X, Y and Z axis:

(8) $$M_{RN}=\left[\begin{array}{ccc} c(\theta_{RN})c(\psi_{RN}) & -c(\phi_{RN})s(\psi_{RN})+s(\phi_{RN})s(\theta_{RN})c(\psi_{RN}) & s(\phi_{RN})s(\psi_{RN})+c(\phi_{RN})s(\theta_{RN})c(\psi_{RN})\\ c(\theta_{RN})s(\psi_{RN}) & c(\phi_{RN})c(\psi_{RN})+s(\phi_{RN})s(\theta_{RN})s(\psi_{RN}) & -s(\phi_{RN})c(\psi_{RN})+c(\phi_{RN})s(\theta_{RN})s(\psi_{RN})\\ -s(\theta_{RN}) & s(\phi_{RN})c(\theta_{RN}) & c(\phi_{RN})c(\theta_{RN}) \end{array}\right]$$

The mapping of the original signals in the rotated coordinates system is obtained through:

(9) $$\left[\begin{array}{c} x_{\text{rot}}\\ y_{\text{rot}}\\ z_{\text{rot}} \end{array}\right]=M_{RN}\;[\begin{array}{c} x_{\text{raw}}\\ y_{\text{raw}}\\ z_{\text{raw}} \end{array}]$$

where {*x_raw_,y_raw_,z_raw_*} and {*x_rot_, y_rot_, z_rot_*} are the raw and transformed acceleration signals respectively. In our particular case XY-axial accelerometers are considered (*z_raw_* = 0̄), thus the rotated signals are eventually defined as:

(10) $$\begin{array}{c} x_{\text{rot}}=\left[c(\theta_{RN})c(\psi_{RN})\right]\;x_{\text{raw}}-\left[c(\phi_{RN})s(\psi_{RN})+s(\phi_{RN})s(\theta_{RN})c(\psi_{RN})\right]\;y_{\text{raw}}\\ y_{\text{rot}}=\left[c(\theta_{RN})s(\psi_{RN})\right]\;x_{\text{raw}}+\left[c(\phi_{RN})c(\psi_{RN})+s(\phi_{RN})s(\theta_{RN})s(\psi_{RN})\right]\;y_{\text{raw}} \end{array}$$

The modeling of the noise associated to a sensor displacement is not as simple as for the rotational case. It does not only depend on the sensor but also the original location, the possible displacement directions and the performed activities. For example, when sensors originally conceived for being attached to the upper part of the extremities (shoulder/thigh) relocates at the end (wrist/ankle), higher acceleration might be measured. An attenuation might be conversely observed when the sensors are displaced the other way around. Nevertheless, this is not always generalizable since it also depends on the particular executed actions. A subject walking may have higher acceleration in the lower arms than in the uppers, but during the execution of some strength-training exercises as push-ups or particular sit-ups the upper arms may suffer higher accelerations. The problem may be even more challenging. So far relative small displacements due to loose attachment or accidental relocations are usually considered. However, the sensors may be erroneously placed on a location completely uncorrelated with the ideal one, introducing an extreme displacement (e.g., the subject switches the ankle sensor with the wrist sensor).

**Figure 2.**
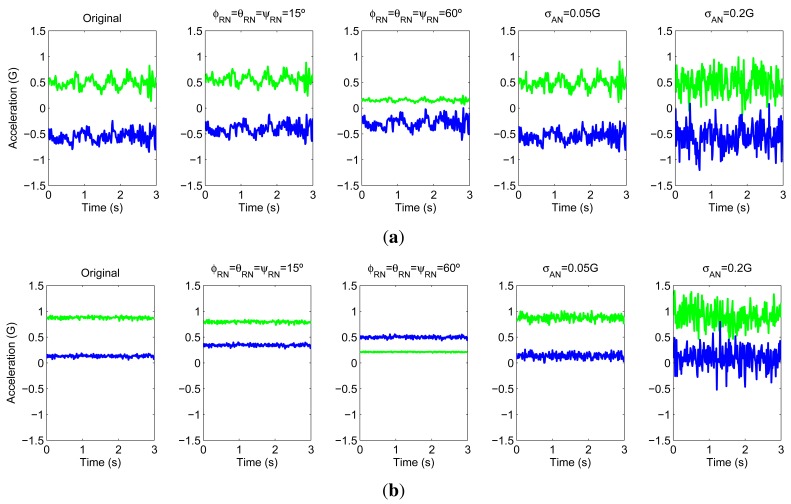
Effect of the rotational and additive noise. X-axis (green) and Y-axis (blue) accelerations recorded through the ankle located sensor when (**a**) walking and (**b**) sitting. Legend: ‘Original’ ≡ raw signals, ‘*φ_rn_* = *θ_rn_* = *ψ_rn_*’ ≡ data with rotational noise, ‘*σ_an_*’ ≡ data with additive noise.

Considering the aforementioned difficulties, and in order to emulate the possible consequences of this anomaly to some extent, we here consider it as part of a more general model which also may represent the effect of uncalibration or battery fails among others arbitrary anomalies. These anomalies usually translate into spurious spikes and attenuations/amplifications altering the sensors readings. A model proposed in [16] based on additive white Gaussian noise characterized by a zero mean and given the strength of the anomaly in the value of the variance $\left(\sigma_{AN}^{2}\right)$ is considered. Even if this model is not as precise as the defined for the rotational noise, the additive noise may model the casuistry of the problem as well as emulate significant changes in the signal space. This may help us to analyze how the intended models behave under different disturbance circumstances. An example of the simulated effect of the described anomalies is depicted in Figure 2.

The particular considered procedure implies the application of the defined noise models to each individual instance. This is only applied to the test instances since the training data is assumed to not comprise such variations (initial design-time trained system). For the subsequent simulations, the rotation angles are arbitrarily varied from 0 to a maximum value ∠*_rn_* for the rotational noise, and coincides with the level defined by *σ_an_* for the additive noise.

### 3.3. Individual Sensor Performance

**Figure 3.**
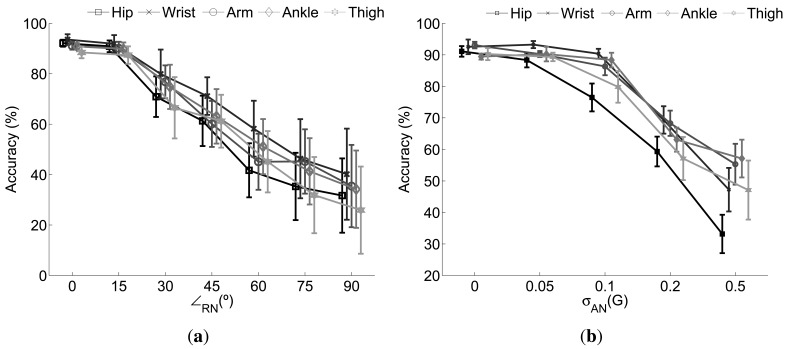
Effect of the (**a**) rotational and (**b**) additive noise when the sensors are separately used. The error bars along the curves correspond to the accuracy standard deviation.

Figure 3 depicts the performance behavior of the single sensor based recognition models under the effect of diverse rotational and additive noise. In both cases the higher the error introduced the lower the accuracy of the recognition system thereby demonstrating poor robustness capabilities when the data of one sole sensor is considered. Particularly for the case of the rotational noise [Figure 3(a)] slight variations (∠*_rn_* ≤ 15°) are normally well tolerated. However, a significant drop is observed for sensor rotations of 30° or more. Clearly, as may be concluded from Equation (10), the effect of the rotation is more notable as higher its value. For example, for *θ_rn_* = 90° *x_rot_* turns to be a scaled version of *y_raw_* with *θ_RN_* and *ψ_RN_* determining the scale factor. Therefore, the signal measured on the X axis has no relation with the measurable signal under ideal circumstances in that very axis. As this example, there are infinite combinations of *φ_rn_*, *θ_rn_* and *ψ_RN_* leading to infinite transformations of the signal space. This normally translates in substantial changes in the feature space which may not be faced by the initial trained systems.

A particularized analysis of the performance of each sensor drives us to conclude the hip and thigh sensors are the less robust to rotations. This is probably because the registered acceleration in those positions is less intense and informative than in the rest, thereby defining more sensitive classification boundaries. In this regard the wrist and ankle sensors are the most robust to rotations as confirmed by the obtained results.

According to the effect of the anomalies modeled through the additive noise, the performance remains practically identical when the level of introduced noise is negligible. That might correspond to a non-significant displacement of the sensor or a inappreciable uncalibration or signal degradation. In any case the monitored signals do not substantially change with respect to the ones recorded on the original setup. However, when the noise level is increased up to 200 *mG* the data distributions are sufficiently altered so the model is unable to face the changes. This translates in a 30% average performance drop with respect to the one obtained in normal circumstances. The drop is further increased to more than 45% when *σ_an_* rises up to 500 *mG*. Again the most sensitive sensors are the hip and the thigh. A similar explanation to the one provided for the rotational noise may be likewise suggested here. Therefore we can in principle state that the hip and the thigh locations are less recommendable when a solution is considered based on just a single sensor.

Finally we also want to stress on the increasing standard deviation values obtained as the noise does. Since the rotation may vary between 0 and the ∠*_rn_* established for each experiment (iteration), different possible abnormal setups are tested thus driving to different results. For the case of the additive noise the standard deviation is a consequence of the stochastic model considered.

### 3.4. Sensor Fusion Performance

**Figure 4.**
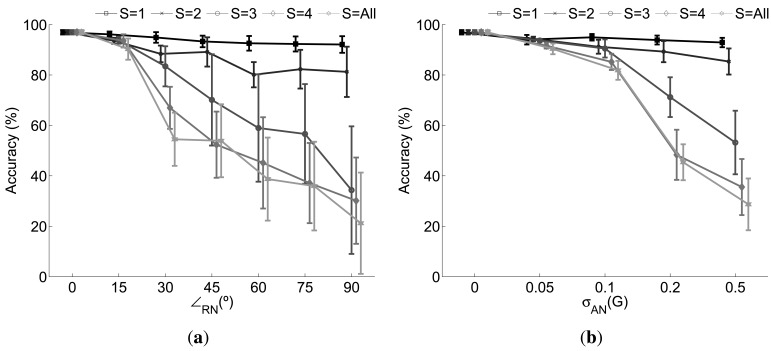
Effect of the (**a**) rotational and (**b**) additive noise when the sensor fusion approach is considered. *S* identifies the number of sensors simultaneously disturbed by the respective noise.

A similar study is presented in Figure 4 for the sensor fusion approach. Now a subset *S* of the sensors is disturbed by the corresponding noise for each case. The affected sensors are randomly selected from one iteration to the next in order to guarantee that different combinations of disturbed sensors are tested, at any rate reflecting what may happen in a real context.

Significant differences may be highlighted with respect to the use of one individual sensor. According to the rotational noise it may be seen the performance remains practically unaltered independent of the level of noise added when one single sensor is affected. This is due to the fact that the decision yielded by the rest of the sensors cope with the failures introduced by the disturbed one, thereby allowing for a performance similar to what is achievable in normal circumstances. The model is also able to satisfactorily overcome the challenge of two rotated sensors. However, since there are more potential situations where the sensors may fail, the probability of coinciding in a misclassification is higher and in consequence the decision provided by the fusion possibly erroneous. This leads to certain cases where the fusion provides a poorer performance. In fact, this is clearly seen when a majority of the sensors are disturbed (*S* ≥ 3), with a decreasing performance which nevertheless overtakes the achieved when a single sensor based recognition system is considered.

The above analysis is also extended for the case of the additive noise. For *S* ≤ 2 the system performs almost perfectly but a significant level of noise on three or more sensors results in an important performance worsening. In any case the results systematically outperform the obtained for the individual case demonstrating the usefulness of the SWNC.

## 4. Discussion

From the analysis of the performance of individual sensors we may conclude that a recognition system based on a single sensor is not a suitable approach but for really slight disturbances. The significance of this statement has been shown to depend on where the device is originally attached. The sensors subject to a limited mobility normally measure lower levels of acceleration, thus being specially indicated for the discrimination between sedentary activities. In such circumstances the static acceleration basically dominates so the effect of the noise is particularly appreciable, especially for the rotations. For example, sensors located on the hip may discriminate between activities such as standing or lying down, but if the sensor is significantly rotated the system may interpret the former activity as the latter and vice versa. The additive noise also further disturbs them. The changes in the data distributions are more relevant since the imposed noise leads to signals completely different to the corresponding original signal space. On the contrary, sensors located on the extremities (primarily the upper ones) have been demonstrated to suffer such variations to a lesser extent. In this regard the wrist sensor is the most reliable of the whole set of analyzed sensors.

Relying on a single sensor may be risky since the reliability really depends on the impact of the disturbances. Consequently, the use of nodes of redundancy is highly recommendable to counteract the effect of these anomalies. Assuming a minority of the sensors are disturbed, independent of the magnitude of the disturbances, the fusion proposed method leads to a highly reliable recognition system which significantly outperforms the results of the individual case. Practically no drop in the performance is suffered when just one sensor is disturbed. The improvement of the reliability and the robustness capabilities nevertheless depends on the intensity of the disturbances when a majority of the sensors are perturbed. In either case, the proposed solution always outperforms the recognition capabilities of the individual systems under any circumstances.

A quantitative comparison with the state-of-the-art approaches is difficult since different setups, datasets and methodologies are considered for each case. In addition to that, not many contributions have been previously presented, as was indicated in Section 1. In any case, here we try to qualitatively compare our proposal with respect to the others. In particular, due to the similarities in the problem addressed, the work of Zappi *et al.* [15] is specifically regarded. Zappi showed that simple fusion models may deal with rotation and faults anomalies when a significant number of nodes (up to 19) is considered. Nevertheless, the performance significantly decreases for reduced sensor networks. In this regard our approach offer a more scalable solution with remarkable results even when just five sensors compose the whole network. Additionally, in [15] the study is just restricted to the upper body extremities, so no analysis is provided with respect to which sensor locations are more sensitive to the considered anomalies. In our work as introduced above, we have demonstrated that the sensors located on the more motion-limited body parts are the most sensitive to the effects of the anomalies. Finally, we also want to highlight that the results shown in [15] correspond to a subject specific (indeed just one) and instance-based setup, with multiple subsequent repetitions of a specific gesture. As a result, the realizations of the considered classes in principle define a more compact signal space, thereby easier reflecting the effect of the anomalies on the behaviour of the classification entities. Our approach has been conversely tested on a more general context, with a higher number of subjects (up to 20) and a more challenging naturalistic context where the effect of the anomalies may be more difficult to encounter.

Since the disturbances might simultaneously affect all the devices in a real case, the problem of the performance worsening is not completely solved. For that reason other additional mechanisms should be possibly considered. One approach is increasing the robustness of the recognition system through defining more robust data distributions. The idea would be to emulate the possible changes in the signal space and include them in order to be learnt during the system training. For example, for the problem associated to the sensor rotations one may think about training the system with artificially modified data for different levels of the considered anomalies. Another alternative would be to collect data for a sufficient number of representative possible rotations, but it may be hard to fully cover all the potential rotations that a sensor may suffer from its original position. This approach would nevertheless reveal more about the impact of other anomalies of complex synthesis as the displacements. According to the suggested fusion model, an interesting approach would be to automatically update the parameters of the model to reduce the impact of the decisions yielded by the disturbed sensors. Nevertheless the task of ascertaining which sensors have been disturbed is very challenging since in principle additional external ground truth information is required to identify them. In either case, and particularly for small sensor networks, the recovery of the “faulty” sensors may be really valuable. In this regard, the use of state-of-the-art adaptive methods like reinforcement learning, active learning or incremental training might be particularly indicated [29].

## 5. Conclusions

Most of the works in activity recognition assume that the setup remains unchanged during runtime, which nevertheless is not a lifelike assumption. In this work we presented an activity recognition methodology which partially deals with the challenging problem of the sensor setup variability. In particular, the effect of the main disturbances that wearable inertial sensors are subject to has been analyzed. The disturbances were synthesized through rotational and additive noise respectively modeling the rotation, displacement or uncalibrations, among other anomalies the sensors may experience in a realistic context.

Sensor anomalies may lead to a significant alteration in the data distribution. Systems trained under the consideration that the devices remain unaltered during runtime have been demonstrated to not suffice when based on a single sensor. The comparison of the robustness of sensors located on different body locations demonstrates that those locations which are more constrained in terms of mobility (such as the hip or thigh) are more sensitive to the considered anomalies.

The use of sensor fusion may compensate such variations. Even when the setup is hardly modified with respect to the originally considered, the fusion enhances the performance achievable through individual sensors in more than 60% at worst conditions when a minority of the sensors are affected. Furthermore, when slight to moderate variations are considered, the fusion can cope with the effect of the disturbances independent of the number of affected sensors. The methodology proposed does not completely suffice to define a reliable recognition system when a majority of the sensors are strongly disturbed. Alternatively, the combination of our method with adaptive techniques might be envisioned for those cases.
